# Comparisons of Severe Maternal Morbidity and Other Adverse Pregnancy Outcomes in Pregnant People With Sickle Cell Disease vs Anemia

**DOI:** 10.1001/jamanetworkopen.2022.54545

**Published:** 2023-02-02

**Authors:** Macy L. Early, Ahizechukwu C. Eke, Alison Gemmill, Sophie Lanzkron, Lydia H. Pecker

**Affiliations:** 1 Division of Hematology, Department of Medicine, Johns Hopkins University School of Medicine, Baltimore, Maryland; 2 Division of Maternal-Fetal Medicine, Department of Gynecology and Obstetrics, Johns Hopkins University School of Medicine, Baltimore, Maryland; 3 Department of Population, Family and Reproductive Health, Johns Hopkins Bloomberg School of Public Health, Baltimore, Maryland; 4 Division of Hematology, Department of Medicine, Johns Hopkins University School of Medicine, Baltimore, Maryland

## Abstract

**Importance:**

Pregnancy in sickle cell disease (SCD) is high risk, but whether prenatal anemia, which is treatable with red blood cell transfusions, is a mediator associated with adverse pregnancy outcomes (APOs) is not known.

**Objective:**

To compare rates and odds of severe maternal morbidity (SMM) and other APOs in pregnancies among individuals with SCD vs those without SCD but with prenatal anemia.

**Design, Setting, and Participants:**

This cross-sectional study was conducted using data from 2012 to 2018 from the National Inpatient Sample, a nationally representative sample of 20% of acute hospital admissions in the United States. All admissions with codes for delivery of a pregnancy among people aged 11 to 55 years were included. Only admissions coded with Black race were included. Data were analyzed from September 2021 through August 2022.

**Exposures:**

Prenatal anemia and SCD.

**Main Outcomes and Measures:**

SMM was tabulated per the Center for Disease Control and Prevention SMM Index alongside other APOs. Multiple logistic regression was used to compare the odds for APOs and risk ratios (RRs) to compare rates of APOs.

**Results:**

Among 764 455 total delivery admissions among patients identified as Black (mean [SD] age at delivery, 27.00 [6.08] years), 3200 deliveries were coded with maternal SCD, 34 808 deliveries were coded with maternal anemia, and 726 447 deliveries were control. Most patients were publicly insured (499 060 [65.4%]). For most outcomes, including SMM and mortality per 10 000 deliveries, the SCD group had higher rates (SMM: 5.9%; 95% CI, 5.1%-6.8%; maternal mortality: 13.0 deaths; 95% CI, 4.9 to 35.0 deaths) than anemia (SMM: 2.1%; 95% CI, 2.0%-2.3%; maternal mortality: 0.9 deaths; 95% CI, 0.3 to 2.8 deaths) or control groups (SMM: 1.1%; 95% CI, 1.0%-1.1%; maternal mortality: 1.2 deaths; 95% CI, 1.0 to 1.5 deaths). SCD (adjusted odds ratio [aOR], 5.51; 95% CI, 4.71-6.45) and anemia groups (aOR, 2.00; 95% CI, 1.84-2.17) had higher adjusted odds of SMM compared with the control group. However, for many complications associated with ischemia or abnormal placentation, CIs of aORs for SCD and anemia groups overlapped (eg, eclampsia: aOR, 2.74; 95% CI, 1.51-4.96 vs aOR, 1.40; 95% CI, 1.08-1.81). For these complications, RRs for SCD vs anemia were between 1.0 and 2.1 (eg, eclampsia: 1.76; 95% CI, 0.93-3.32). For complications associated with thrombosis or SCD-specific pathologies, rates and aORs were greater for the SCD vs anemia group. For these complications, RRs were between 3.70 and 10.90. For example, rates of acute respiratory distress syndrome, including acute chest syndrome, were 56 of 3144 deliveries (1.8%) vs 122 of 34 686 deliveries (0.4%), and the RR was 4.99 (95% CI, 3.65-6.84).

**Conclusions and Relevance:**

This study found that risks associated with prenatal anemia and SCD were similar for many APOs, especially those associated with ischemia and abnormal placentation, suggesting that prenatal anemia may be a mediator associated with pregnancy risk in individuals with SCD.

## Key Points

**Question:**

Are rates of severe maternal morbidity different among pregnant people with sickle cell disease (SCD) compared with those with prenatal anemia?

**Findings:**

In this cross-sectional study of 764 455 deliveries among patients identified as Black, deliveries among individuals with SCD and anemia had similar odds of complications that are associated with ischemia and cardiovascular stress, such as kidney failure, shock, and mechanical ventilation. Deliveries among individuals with SCD had significantly higher odds of thrombophilic complications, including cerebrovascular events and venous thromboembolism.

**Meaning:**

These findings suggest that prenatal anemia, which is modifiable by transfusion, may be a mediator associated with some of the increased odds of severe maternal morbidity in SCD.

## Teaser

This cross-sectional study compares rates and odds of severe maternal morbidity and other adverse pregnancy outcomes among Black pregnant individuals with sickle cell disease vs anemia.

## Introduction

Pregnancy in people with sickle cell disease (SCD) is high risk. There are 50 years of data finding several-fold increased rates of severe pregnancy complications and maternal mortality in pregnancies among individuals with SCD.^1,2,3^ The pathophysiology of SCD is complex and characterized by inflammation, endothelial dysfunction, vaso-occlusion, and anemia.^4^ Anemia may be a mediator associated with some portion of the risk for severe maternal morbidity (SMM) and other adverse pregnancy outcomes (APOs) in pregnancy among individuals with SCD (eFigure in Supplement 1).

In the population of pregnant individuals without SCD, anemia is a recognized risk factor associated with APOs.^5,6^ Prenatal anemia itself is associated with an especially high increase in risk of hypertensive disorders of pregnancy (HDP), intrauterine growth restriction, and preterm delivery.^5^ In pregnancy, anemia is defined by trimester as hemoglobin levels less than 11 g/dL in the first and third trimesters and less than 10.5 g/dL in the second trimester (to convert to grams per liter, multiply by 10.0).^7^ Using this definition, nearly all pregnant people with SCD have prenatal anemia.^8,9^ However, despite recent interest in the association of anemia with SCD outcomes, the association of prenatal anemia with complications in pregnancies among individuals with SCD have not been explored to our knowledge.^10^ Understanding the association between anemia and APOs in pregnancies among individuals with SCD is essential because anemia can be treated with red blood cell transfusions. However, current indications for transfusion in pregnancies among individuals with SCD do not include acute or chronic anemia.^11^

The primary objective of this study was to use the US-based National Inpatient Sample (NIS) to directly compare rates and odds of SMM and other APOs in pregnancies among individuals with SCD vs those without SCD but with prenatal anemia. Our goal was to explore the potential role of anemia as a mediator associated with outcomes in pregnancies among individuals with SCD.

## Methods

This cross-sectional study was approved by the Johns Hopkins Institutional Review Board (IRB). Informed consent was waived by the Johns Hopkins IRB due to the use of deidentified data. The study adheres to the Strengthening the Reporting of Observational Studies in Epidemiology (STROBE) reporting guideline.

### Data Set

This study used data from the 2012 to 2018 NIS, which includes all years since the NIS 2012 sampling frame redesign. The NIS is a 20% stratified sample of all discharges from US acute care hospitals. It is managed by the Agency for Healthcare Research and Quality and was previously described.^12^ As of 2012, the sample reflects 97% of the US population. NIS admissions are fully deidentified.

### Inclusion Criteria

We identified admissions involving the delivery of pregnancies by *International Classification of Diseases, Ninth Revision* (*ICD-9*) and *International Statistical Classification of Diseases and Related Health Problems, Tenth Revision* (*ICD-10*) codes. We included deliveries to people who were aged 11 to 55 years and coded as having Black race. In the US context, most people with SCD are identified as having Black race.^13^ Inequities associated with Black race contribute to adverse maternal outcomes in the United States.^14^ However, there are not sufficient data to guide whether Black race should be treated as a mediator, confounder, or covariate. Instead, we elected to include only pregnant people with Black race in our comparator groups to control for the complex effects of racial disparities. In the NIS, the variable titled race is drawn from discharge records from source hospitals. The method by which maternal racial classification was determined for each pregnancy varied based on hospital protocols.

We excluded admissions for which race or diagnosis data were missing and admissions containing codes for miscarriage, which are often managed in the outpatient setting and cannot be reliably studied using an inpatient database. We further excluded deliveries with codes for multifetal gestation, which confound interpretation of SMM and anemia. The *ICD-9* and *ICD-10* codes used to identify and classify admissions are included in eTable 1 in Supplement 1.

### Outcomes

We quantified APOs using *ICD-9* and *ICD-10* codes. We used the Centers for Disease Control and Prevention (CDC) SMM Index, which includes 21 highly morbid obstetric complications, as our primary outcome measure. Due to confounding by SCD, we excluded 2 SMM outcomes from the analysis: transfusion and sickle cell vaso-occlusive crisis. Final analytic models included 19 of 21 SMM outcomes considered individually and in a composite SMM variable (eTable 1 in Supplement 1). The *ICD-9* and *ICD-10* codes listed in the CDC SMM Index have an 86% positive predictive value for identifying SMM in administrative data.^15^ Although transfusion was excluded in the SMM analysis, it was analyzed as an independent secondary outcome. Additional APOs analyzed were gestational hypertension, preeclampsia, placental abruption, preterm premature rupture of membranes, cesarean delivery, instrumented vaginal delivery, preterm delivery, peripartum infection (urinary tract infection, endomyometritis, wound infection, or sepsis), postpartum hemorrhage, intrauterine growth restriction, and intrauterine fetal demise.

### Defining Anemia

In defining our group for deliveries among individuals with anemia, we aimed to identify patients with prenatal anemia and exclude those who developed anemia at delivery due to acute blood loss. The appropriate combination of *ICD-9* and *ICD-10* codes to use in identifying our target population was not immediately clear, so we compared 3 candidate definitions with published features of a population of 10 000 pregnant people with prenatal anemia from a retrospective registry.^6^ The first candidate definition was anemia of pregnancy: this inclusive definition captured all deliveries coded with any anemia of pregnancy code or subcode. We suspected high sensitivity of this broad definition and low specificity because people with acute peripartum blood loss anemia were also captured with this definition. The second candidate definition was deficiency anemia only: iron deficiency anemia is the most common cause of prenatal anemia. This definition captured all deliveries explicitly coded with iron, folate, or vitamin B12 deficiency. We suspected low sensitivity and high specificity for this definition. However, we were concerned about biasing our group toward individuals with deficiency anemia that began before conception, who may be at higher risk for obstetric outcomes than the general population with prenatal anemia. The third was a mixed definition, for which we included deliveries coded with the general anemia complicating pregnancy code unless those deliveries were also coded with postpartum hemorrhage. We also included deliveries coded with a deficiency anemia code or a code for prenatal anemia of pregnancy regardless of whether the delivery also contained codes for postpartum hemorrhage. Results comparing these approaches are in eTable 2 in Supplement 1. The mixed definition was selected as the anemia definition for this study.

### Definition of Analysis Groups

We used 3 analysis groups in our study. The first was the SCD deliveries group, which this consisted of admissions with at least 1 SCD diagnosis code and no sickle cell trait codes. This approach has a greater than 90% positive predictive value for identifying individuals with SCD.^16^ Codes for *ICD-9* and *ICD-10* unreliably identify SCD genotype,^17^ so SCD deliveries were not subdivided. The second was the anemia deliveries group, which consisted of admissions without an SCD diagnosis code that met the mixed definition of anemia. The third was the control deliveries group, which consisted of admissions with neither an SCD diagnosis code nor codes meeting the mixed definition of anemia.

### Statistical Analysis

#### Primary Analyses

Descriptive characteristics and rates of SMM and other APOs in anemia and control delivery groups were compared with those in the SCD delivery group using *t* or χ^2^ tests. We compared odds of SMM and other APOs in SCD and anemia delivery groups vs the control delivery group using multiple logistic regression models that estimated adjusted odds ratios (aORs) and 95% CIs. The eFigure in Supplement 1 depicts our hypothesis of the pathway by which anemia may be associated with APOs in pregnant individuals with SCD. We included patient age, public insurance status, and income quartile by zip code and hospital volume, teaching status, ownership (government, private nonprofit, or private investor owned), and regional location as confounders or covariates in our multiple logistic regression models.

#### Secondary Analyses

In secondary analyses, we assessed the contributions of anemia to SMM and other APOs among pregnant people with SCD. We used rates to calculate the relative risk (RR) of outcomes in SCD vs anemia delivery groups.

Analyses were performed with Stata SE statistical software version 17.0 (StataCorp).^18^ Missing data were handled with listwise deletion for each model in accordance with standards issued by data set administrators and implemented in previous studies using the NIS.^19^ Information about missing data is included in eTable 3 in Supplement 1. Statistical tests were survey weighted. To minimize the risk of false discovery, we applied the Benjamini and Yekutieli correction^20^ across analyses and used a tolerable false discovery rate of less than 5%. All reported *P* values are 2-sided and corrected; when a comparison is reported as significant, the *P* value is less than .05 after multiple comparisons correction. Data were analyzed from September 2021 to August 2022.

## Results

### Demographic and Hospital Characteristics for Deliveries

The sample included 764 455 deliveries among Black birthing people (mean [SD] age at delivery, 27.10 [6.08] years). There were 3200 deliveries in the SCD group, 34 808 deliveries in the anemia group, and 726 447 deliveries in the control group. Demographic, hospital, and delivery admission characteristics are described in Table 1.^21^ Most patients were publicly insured (499 060 patients [65.4%]). There was no difference in mean (SD) age at delivery between SCD and anemia groups (27.03 [5.77] years vs 26.80 [6.00] years) or in the proportion of patients older than age 35 years (403 patients [12.6%] vs 4299 patients [12.4%]). Income distributions by zip code were similar across groups, although the anemia group had a higher proportion of people who resided in zip codes with median incomes in the lowest quartile. Most patients in each group were on public insurance, although the proportion was lower among patients in the control group. In each group, most deliveries occurred in large, urban teaching hospitals, but the highest proportion of deliveries in the SCD group occurred in this setting. Deliveries in SCD and anemia groups had the same rate of multiple gestation, which was higher than the rate in the control group. Deliveries in the SCD group had the longest length of stay.

**Table 1.** Characteristics of Delivery Admissions

Characteristic	Deliveries, No. (%) (N = 764 455)			*P* value, anemia vs SCD	*P* value, control vs SCD
	SCD (n = 3200)	Anemia (n = 34 808)	Control (n = 726 447)		
Age, mean (SD), y	27.03 (5.77)	26.80 (6.00)	27.11 (6.09)	.60	.51
Patients aged ≥35 y	403 (12.6)	4299 (12.4)	106 487 (13.3)	.52	.26
Household income by zip code, $					
1-24 999	1530 (48.7)	18 375 (53.4)	354 588 (49.5)	<.001	.39
25 000-34 999	757 (24.1)	7599 (22.1)	164 996 (23.0)		
35 000-44 999	552 (17.6)	5469 (15.9)	122 961 (17.2)		
≥45 000 or more	306 (9.7)	3002 (8.7)	74 112 (10.3)		
Public insurance	2232 (69.9)	24 219 (69.7)	472 609 (65.2)	.22	<.001
Teaching status, urban					
Teaching	2583 (80.7)	26 969 (77.5)	528 057 (72.7)	<.001	<.001
Nonteaching	528 (16.5)	5779 (16.6)	156 114 (21.5)		
Rural	89 (2.8)	2060 (5.9)	42 246 (5.8)		
Hospital volume^a^					
Small	336 (10.5)	5579 (16.0)	97 501 (13.4)	<.001	<.001
Medium	893 (27.9)	10 471 (30.1)	232 200 (32.0)		
Large	1971 (61.6)	18 758 (53.9)	396 746 (54.6)		
Multiple gestation	130 (4.1)	1421 (4.1)	17 670 (2.4)	.85	<.001
Length of stay, mean (SE), d	4.6 (0.10)	3.4 (0.03)	2.9 (0.01)	<.001	<.001

Abbreviation: SCD, sickle cell disease.

^a^ Categorization of hospital volume depends on a hospital's teaching status and geographic location. Detailed information is available at the Healthcare Utilization Project website.^21^

### Rates of SMM and Mortality

We measured the prevalence of SMM in each group; eTable 4 in Supplement 1 shows rates of each SMM complication. The rate of composite SMM among deliveries was 5.9% (95% CI, 5.1%-6.8%) in the SCD group, 2.1% (95% CI, 2.0%-2.3%) in the anemia group, and 1.1% (95% CI, 1.0%-1.1%) in the control group. Maternal mortality was highest in the SCD group and lowest in the anemia group. The death rate per 10 000 deliveries was 13.0 deaths (95% CI, 4.9 to 35.0 deaths) in SCD vs 0.9 deaths (95% CI, 0.3 to 2.8 deaths) in anemia and 1.2 deaths (95% CI, 1.0 to 1.5 deaths) in control groups.

### Odds of SMM and Adverse Fetal Outcomes

**Figure 1.**
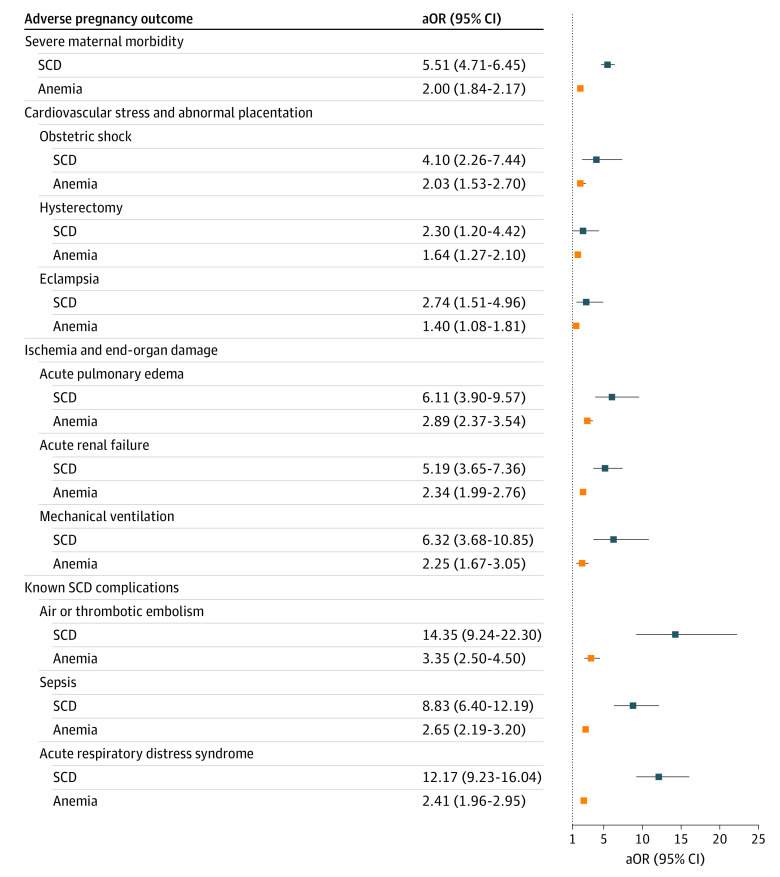
Adjusted Odds of Severe Maternal Morbidity Adjusted odds ratios (aORs) include controls for age, income quartile by zip code, and public insurance status for patients and teaching status and size for hospitals.

Adjusted odds of SMM and other APOs were calculated using multivariable models that included age, public insurance status, and income quartile by zip code for patients and geographical region, bed size, and teaching status for hospitals (Table 2, Figure 1). We compared aORs vs control deliveries for the SCD group with those of the anemia group. For all complications, aORs were greater in the SCD than the anemia group. For example, for SMM, the aOR was 5.51 (95% CI, 4.71-6.45) for the SCD and 2.00 (95% CI, 1.84-2.17) for the anemia group. CIs of aORs for SCD and anemia groups overlapped for obstetric shock (4.10; 95% CI, 2.26-7.44 vs 2.03, 95% CI 1.53-2.70), hysterectomy (2.30; 95% CI, 1.20-4.42 vs 1.64; 95% CI, 1.27-2.10), and eclampsia (2.74; 95% CI, 1.51-4.96 vs 1.40; 95% CI, 1.08-1.81), which are complications associated with cardiovascular stress or abnormal placentation. Lower bounds of SCD aOR CIs were approached by upper bounds of anemia aOR CIs for acute kidney failure (5.19; 95% CI, 3.65-7.36 vs 2.34; 95% CI, 1.99-2.76), mechanical ventilation (6.32; 95% CI, 3.68-10.85 vs 2.25; 95% CI, 1.67-3.05), and acute pulmonary edema (6.11; 95% CI, 3.90-9.57 vs 2.89; 95% CI, 2.37-3.54), which are associated with ischemia and end-organ damage. SCD aORs were greater than anemia aORs for air or thrombotic embolism (14.35; 95% CI, 13.50-16.31 vs 3.35; 95% CI, 2.50-4.50), sepsis (8.83; 95% CI, 6.40-12.19 vs 2.65; 95% CI, 2.19-3.20), and acute respiratory distress syndrome (12.17; 95% CI, 9.23-16.04 vs 2.41; 95% CI, 1.96-2.95), all of which are known complications associated with SCD.

**Table 2.** Odds of SMM and Adverse Fetal Outcomes

Outcome	aOR (95% CI)	
	SCD vs control	Anemia vs control
SMM	5.51 (4.71-6.45)	2.00 (1.84-2.17)
Cardiovascular stress or abnormal placentation		
Obstetric shock	4.10 (2.26-7.44)	2.03 (1.53-2.70)
Hysterectomy	2.30 (1.20-4.42)	1.64 (1.27-2.10)
Eclampsia	2.74 (1.51-4.96)	1.40 (1.08-1.81)
Ischemia or end-organ damage		
Acute pulmonary edema	6.11 (3.90-9.57)	2.89 (2.37-3.54)
Acute kidney failure	5.19 (3.65-7.36)	2.34 (1.99-2.76)
Mechanical ventilation	6.32 (3.68-10.85)	2.25 (1.67-3.05)
Known complications associated with SCD		
Air or thrombotic embolism	14.35 (13.50-16.31)	3.35 (2.50-4.50)
Sepsis	8.83 (6.40-12.19)	2.65 (2.19-3.20)
Acute respiratory distress syndrome	12.17 (9.23-16.04)	2.41 (1.96-2.95)
Adverse fetal outcome		
Intrauterine growth restriction	2.15 (1.89-2.44)	1.08 (1.03-1.15)
Preterm delivery	1.14 (1.00-1.30)	0.97 (0.93-1.02)
Intrauterine fetal demise	1.42 (1.09-1.86)	0.79 (0.71-0.88)

Abbreviations: aOR, adjusted odds ratio; SCD, sickle cell disease; SMM, severe maternal morbidity.

Adjusted odds of fetal complications are shown in Table 2. Compared with control deliveries, the odds of intrauterine growth restriction were increased in SCD (aOR 2.15; 95% CI, 1.89-2.44) and anemia (aOR 1.08; 95% CI, 1.03-1.15), although the increase in odds was substantially greater in the SCD group. Neither group had increased adjusted odds of preterm delivery. The risk of intrauterine fetal demise was increased in the SCD group (aOR 1.42; 95% CI, 1.09-1.86) but decreased in the anemia group (aOR 0.79; 95% CI, 0.71-0.88).

### Odds of Other APOs in SCD and Anemia Groups

We assessed 3 categories of additional APOs: HDP, delivery-associated complications, and hematologic and immunologic complications. Rates of each outcome are reported in Table 3. The rate of any HDP was 511 deliveries (15.6%) for the SCD group, 5296 deliveries (14.8%) for the anemia group, and 85 769 deliveries (12.1%) for the control group. The most prevalent delivery-associated complication was cesarean delivery (SCD: 1238 deliveries [38.5%]); anemia: 13 410 deliveries [37.4%]; control: 236 788 deliveries [33.4%]), and the most prevalent hematologic and immunologic complications included transfusion (SCD: 648 deliveries [19.8%]; anemia: 1986 deliveries [5.3%]; control: 11 433 deliveries [1.6%]) and any peripartum infection (SCD: 388 deliveries [12.0%]; anemia: 2533 deliveries [7.2%]; 36 511 deliveries [5.2%]).

**Table 3.** Prevalence of Adverse Pregnancy Outcomes

Outcome	No. (%) (N = 764 455)		
	SCD (n = 3200)	Anemia (n = 34 808)	Control (n = 726 447)
Hypertensive disorders of pregnancy			
Any hypertensive disorder	511 (15.6)	5296 (14.8)	85 769 (12.1)
Preeclampsia	372 (11.3)	3234 (8.9)	50 278 (7.1)
Eclampsia	11 (0.4)	68 (0.2)	930 (0.1)
Delivery-associated complication			
Cesarean delivery	1238 (38.5)	13 410 (37.4)	236 788 (33.4)
Postpartum hemorrhage	185 (5.7)	1320 (3.8)	24 712 (3.5)
Instrumented delivery	101 (3.2)	1280 (3.7)	30 182 (4.3)
Preterm prelabor rupture of membranes	105 (3.0)	1298 (3.6)	26 572 (3.8)
Abruption	44 (1.4)	643 (1.8)	9996 (1.4)
Hematology or immunologic complication			
Transfusion	648 (19.8)	1986 (5.3)	11 433 (1.6)
Any peripartum infection	388 (12.0)	2533 (7.2)	36 511 (5.2)
Venous thromboembolism	37 (1.1)	64 (0.3)	466 (0.1)

Abbreviation: SCD, sickle cell disease.

#### Hypertensive Disorders of Pregnancy

Comparing the SCD with anemia group, odds of any HDP were not increased in deliveries among individuals with SCD (aOR 1.05, 95% CI 0.95-1.16). Odds of preeclampsia and eclampsia were increased in the SCD compared with the anemia group (preeclampsia: aOR 1.26, 95% CI 1.12-1.42; eclampsia: aOR 1.99, 95% CI 1.05-3.77).

#### Delivery-Associated Complications

Comparing the SCD with the anemia group, there were not increased odds in deliveries among individuals with SCD for cesarean delivery (aOR 1.05, 95% CI 0.97-1.14), instrumented delivery (aOR 0.88, 95% CI 0.71-1.09), preterm prelabor rupture of membranes (aOR 0.81, 95% CI 0.65-1.01), or placental abruption (aOR 0.75, 95% CI 0.54-1.04). The SCD group had higher odds of postpartum hemorrhage compared with the anemia group (aOR 1.52, 95% CI 1.29-1.79).

#### Hematologic and Immunologic Complications

Odds in the anemia group were increased compared with the control group. However, the SCD group had significantly higher odds than the anemia group of transfusion (aOR 4.40, 95% CI 3.96-4.89), peripartum infection (aOR 1.68, 95% CI 1.49-1.90), and venous thromboembolism (aOR 5.90, 95% CI 3.83-9.10).

### Anemia as Mediator Associated With Pregnancy Complication in Individuals With SCD

**Figure 2.**
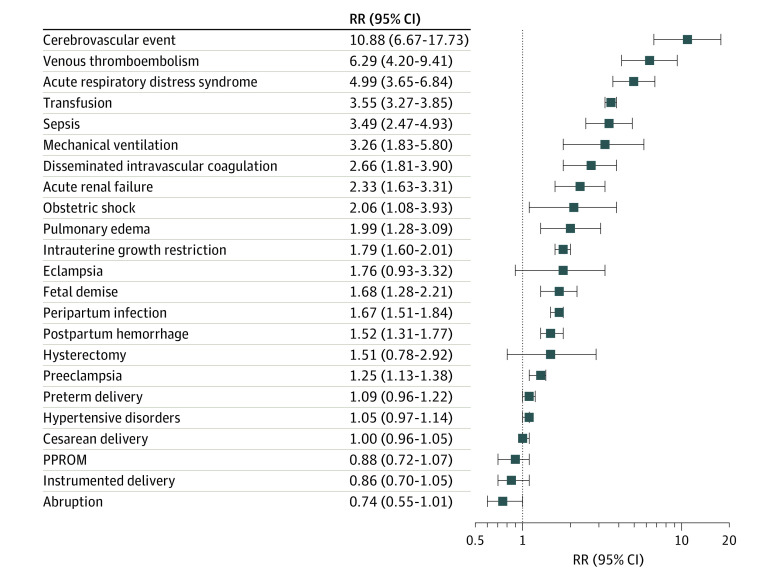
Risk Ratios (RRs) of Adverse Pregnancy Outcomes for Sickle Cell Disease vs Anemia Groups Cerebrovascular event includes central venous thrombosis, stroke, or transient ischemic attack. PPROM indicates preterm premature rupture of membrane.

For most obstetric complications, the highest risk was in the SCD group (Table 2). However, in many cases the rates of adverse outcomes in anemia deliveries approached the rates in SCD deliveries. Risk ratios (RRs) of SMM and APOs, comparing rates in SCD vs anemia groups, are shown in Figure 2. For complications with a documented association with SCD pathophysiology, such as cerebrovascular events, venous thromboembolism, acute respiratory distress syndrome, and transfusion, RRs were high, ranging from 3.55 (95% CI, 3.27-3.85) for transfusion to 10.88 (95% CI, 6.67-17.73) for cerebrovascular events. For complications associated with abnormal placentation and ischemia, such as shock, intrauterine growth restriction, HDP, and hysterectomy, RRs were much smaller, falling between 1.05 (95% CI, 0.97-1.14) for any HDP and 2.33 (95% CI, 1.63-3.31) for acute kidney failure.

## Discussion

In this cross-sectional study of a nationally representative sample, the risks in Black individuals with SCD and Black individuals with anemia were similar for a subset of APOs, including certain SMM outcomes. SCD and anemia delivery groups had similar risks for complications associated with ischemia and cardiovascular stress, such as acute kidney failure, shock, and mechanical ventilation. SCD and anemia groups also had similar risks for complications associated with abnormal placentation, such as HDP and intrauterine growth restriction. For other complications, the risks in the SCD group were several-fold higher than in the anemia group. Complications for which SCD deliveries had distinctly higher risks were thrombophilic, including cerebrovascular events and VTE.

These data preliminarily suggest that prenatal anemia may be a mediator associated with many APOs in pregnancy among individuals with SCD. Our findings align with existing mechanistic evidence. Prenatal anemia predisposes to ischemia, which is associated with end-organ damage and abnormal angiogenesis, and ultimately placental malformation.^5^ Pregnant people with anemia are susceptible to ischemic insults in the setting of blood loss or vasodilation due to lower red blood cell reserves.^6^ Moreover, adaptations to anemia are associated with increased cardiovascular stress and decreased cardiovascular reserve, which may limit pregnant people’s ability to adapt to pregnancy.^22,23^ These mechanisms may explain increased rates of kidney failure, shock, and hysterectomy in SCD and anemia groups in our study. Placentas from anemia-affected pregnancies demonstrate abnormal vascular patterns and errant expression of angiogenic signaling molecules, including vascular endothelial growth factor, placental growth factor, and endothelial nitric oxide synthase.^24^ Similar derangements in angiogenesis have been identified in people with SCD,^25^ and these mechanisms could explain the increased rates of placentally mediated APOs, including HDP and intrauterine growth restriction, in deliveries among individuals with SCD and anemia in this study.

The potential association of anemia with pregnancy outcomes among individuals with SCD compels attention. In the population without SCD, there is evidence of long-lasting harms associated with prenatal anemia.^5,6,26,27^ Prenatal maternal anemia was associated with neurodevelopmental disorders in children who were exposed.^27^ These risks are not yet defined in SCD.^28^ Prenatal anemia is modifiable through transfusion. However, neither acute nor chronic anemia is currently an indication for transfusion in pregnant individuals with SCD.^11^ Extant studies of transfusion in pregnant individuals with SCD are mostly observational and difficult to summarize due to variations in transfusion timing and methods.^11^ However limited, preliminary data suggest that prophylactic transfusion may be associated with improved maternal and fetal outcomes, especially for people with high-risk SCD or obstetric features. However, what constitutes high risk is not yet defined.^29^

We observed lower mortality rates among deliveries by Black individuals with anemia than among those by Black individuals in the control group. Further study may be needed to investigate the association of anemia with modifications in disparities associated with racism. Additionally, mortality was a rare outcome. It is possible that prenatal anemia was not sufficient for an association with mortality in an otherwise-healthy pregnant person. Pregnant people with SCD have prenatal anemia and chronic organ damage, which are associated with increased risk for complications, such as venous thromboembolism. Deliveries among individuals with SCD in our study had remarkably increased risk for these complications.

### Strengths and Limitations

To our knowledge, this is one of few papers to compare pregnancy among individuals with SCD vs pregnancies among individuals with anemia. Our findings suggest that anemia may be a risk factor associated with maternal morbidity and a mediator associated with other APOs in pregnancy. However, this study was limited by our use of administrative data. We selected an administrative definition of prenatal anemia that yielded a sample consistent with previously published data.^6^ We likely undercounted the true population of pregnant people with prenatal anemia because we excluded those coded with postpartum hemorrhage if the admission did not also include a code specifically indicating prenatal anemia. No data are available describing the positive predictive value of *ICD-9* or *ICD-10* codes in identifying antenatal anemia. SCD status may be miscoded for some pregnancies. However, previous studies report greater than 90% positive predictive values using the method we used to identify patients,^16^ and the large size of the data set may mitigate the association of misclassification with changes in results. Given that laboratory data were not available in the data set, we could not stratify by degree of anemia or exclude pregnant people with SCD who had hemoglobin levels greater than 10 g/dL; this may be a subject for future research.

As an administrative data set, the NIS is a limited research tool. We could not include other variables, such as parity, obesity, or tobacco use, in our analyses. We could not follow up pregnancies across admissions given that NIS admissions are not linked to individuals. The NIS data set does not capture out-of-hospital births, which may include less complicated pregnancies, and excludes admissions to federal hospitals like the Veterans Affairs system. However, together, these groups are expected to account for approximately 1.5% of deliveries in the US.^30,31^ Despite its limitations, the NIS draws from most births in the US and provides unmatched sample size and generalizability, providing important hypothesis-generating information for future studies.

## Conclusions

In this cross-sectional study using a nationally representative administrative data set of APOs among people with anemia vs people with SCD, we identified striking similarities in odds of many APOs between pregnancies among individuals with prenatal anemia and those with SCD. This was especially true for APOs for which ischemia, cardiovascular stress, or abnormal placentation are known to be mediators associated with risk. These findings preliminarily suggest that prenatal anemia may be a mediator associated with some of the risk for SMM and APOs in pregnant individuals with SCD. Prenatal anemia is modifiable through transfusion, and clearer indications for transfusion in pregnancies among individuals with SCD are needed. More work is needed to investigate whether a direct, causal link between anemia and APOs exists in pregnancies among individuals with SCD given that this observational study can make no causal conclusions.

## Notes

Supplement 1. **eFigure.** Proposed Pathway by Which Anemia Is Associated With Pregnancy Outcomes in Individuals With Sickle Cell Disease **eTable 1.** Codes Used to Identify Independent and Dependent Variables **eTable 2.** Comparing Code Definitions for Prenatal Anemia vs Published Data **eTable 3.** Proportion of Missing Data by Variable **eTable 4.** Survey-Weighted Rates of Composite Severe Maternal Morbidity and Component Events in Delivery Admissions by Group

Supplement 2.
Data Sharing Statement
