# Supplementary material for: Comparisons of Severe Maternal Morbidity and Other Adverse Pregnancy Outcomes in Pregnant People With Sickle Cell Disease vs Anemia
Source: JAMA Netw Open. 2023 Feb 2;6(2):e2254545. doi: 10.1001/jamanetworkopen.2022.54545 (PMC9896269; doi:10.1001/jamanetworkopen.2022.54545)
Supplement: Supplement 1. — eFigure. Proposed Pathway by Which Anemia Is Associated With Pregnancy Outcomes in Individuals With Sickle Cell Disease eTable 1. Codes Used to Identify Independent and Dependent Variables eTable 2. Comparing Code Definitions for Prenatal Anemia vs Published Data eTable 3. Proportion of Missing Data by Variable eTable 4. Survey-Weighted Rates of Composite Severe Maternal Morbidity and Component Events in Delivery Admissions by Group [file jamanetwopen-e2254545-s001.pdf]

Supplemental Online Content

Early ML, Eke AC, Gemmill A, Lanzkron S, Pecker LH. Comparisons of severe maternal morbidity and other adverse pregnancy outcomes in pregnant people with sickle cell disease vs anemia. *JAMA Netw Open.* 2023;6(2):e2254545.
doi:10.1001/jamanetworkopen.2022.54545

eFigure. Proposed Pathway by Which Anemia Is Associated With Pregnancy Outcomes in Individuals With Sickle Cell Disease
eTable 1. Codes Used to Identify Independent and Dependent Variables
eTable 2. Comparing Code Definitions for Prenatal Anemia vs Published Data
eTable 3. Proportion of Missing Data by Variable
eTable 4. Survey-Weighted Rates of Composite Severe Maternal Morbidity and Component Events in Delivery Admissions by Group

This supplemental material has been provided by the authors to give readers additional information about their work.

© 2023 Early ML et al. *JAMA Network Open.*

eFigure. Proposed Pathway by Which Anemia Is Associated With Pregnancy Outcomes in Individuals With Sickle Cell Disease

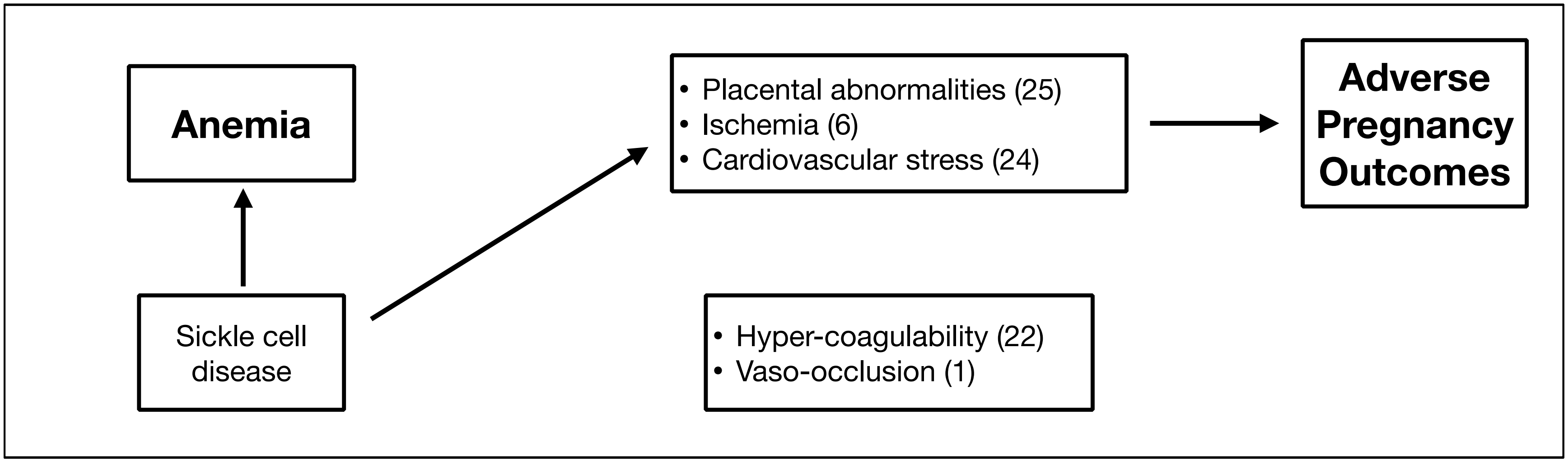

Anemia is associated with adverse pregnancy outcomes. In response to ischemia, abnormal angiogenesis occurs, which, in pregnant people, drives abnormal placental formation and maturation. Ischemia from acute or chronic anemia damages organ function. In a state of chronic anemia, cardiovascular adaptation occurs to increase oxygen delivery to tissues, which is a source of cardiovascular stress. The majority of pregnant people with SCD meet the definition of anemia of pregnancy. In addition to anemia, the chronic hemolysis of SCD causes chronic inflammation and endothelial dysfunction, which drive a hyper-coagulable state and trigger vast-occlusive crises.

© 2023 Early ML et al. *JAMA Network Open.*

eTable 1. Codes Used to Identify Independent and Dependent Variables

Variable	ICD-9	ICD-10
**Descriptive variables**		
Delivery admissions	DX: V27, 650 – 669<br>PR: 72-75	DX: O60 – O82, Z37-Z38<br>PR: 10D07Z3 – 10D07Z8, 10D00Z0 – 10D00Z2, 10E0XZZ, 0DQR0ZZ, 0HQ9XZZ, 0KQM0ZZ, 0W8NXZZ
**Exposures**		
Sickle cell disease	282·60 – 282·69 or 282·41 – 282·42	D57·0 – D57·2*, D57·4*, D57·8*
Anemia complicating pregnancy	648·2*	O99·0*
Antenatal anemia only	648·23	O99·011, O99·012
Deficiency anemia	280*, 281*	D50* D51* D52* D53*
**Pregnancy outcomes**		
Acute heart failure[a]	997·1	I97·12*, I97·13*, I19·71*
Acute myocardial infarction	410·*	I21·01, I21·02, I21·09, I21·11, I21·19, I21·21, I21·29, I21·3, I21·4, I21·9
Acute renal failure[a]	584·5, 584·6, 584·7, 584·8, 584·9, 669·3*	N17·0, N17·1, N17·2, N17·8, N17·9, O90·4
Adult respiratory distress syndrome[a]	518·5*, 518·81, 518·82, 518·84, 799·1	J80, J95·1, J95·2, J95·3, J95·821, J95·922, J96·00, J96·01, J96·02, J96·20, J96·21, J96·22, R09·2
Air or thrombotic embolism[a]	415·1*, 673·0*, 673·2*, 673·3*, 673·8*	I26·*, O88·0*, O88·2*, O88·3*, O88·8*
Amniotic fluid embolism[a]	673·1*	O88·11*, O88·12, O88·13
Aneurysm[a]	441·*	I71·00-I71·03, I71·1, I71·2, I71·3, I71·4, I71·5, I71·6, I71·8, I71·9, I79·0
Cardiac arrest[a]	427·41, 427·42, 427·5	I46·2, I46·8, I46·9, I49·0*
Cerebrovascular event[a]	430·0 – 437·99, 671·5*, 674·0*, 997·02	I60 – I68·8, O22·51, O22·52 I97·81*, I97·82*, O87·3
Cesarean delivery	DX: 669·7<br>PR: 74·0, 74·1, 74·2, 74·4, 74·9*	DX: O82*, O75·82, z3801, z3831, z3862, z3864, z3866, z3869<br>PR: 10D00Z0-10D00Z1
Conversion of cardiac rhythm[a]	99·6*	5A2204Z, 5A12012
Disseminated intravascular coagulation[a]	286·6, 286·9, 666·3*	D65, D68·8, D68·9, O72·3
Eclampsia[a]	642·6*	O15·0*, O15·1, O15·2, O15·9
Hypertensive disorder of pregnancy, composite	642·3* 642·4* 642·5* 642·6* 642·7*	O11* O13* O14* O15*
Hysterectomy[a]	PR: 68·3* - 68·9*	PR: 0UT90ZZ, 0UT94ZZ, 0UT97ZZ, 0UT98ZZ, 0UT9FZZ

© 2023 Early ML et al. *JAMA Network Open.*

Instrumented vaginal delivery	DX: 669·5<br>PR: 72*	PR: 10D07Z3, 10D07Z4, 10D07Z5, 10D07Z6, 10D07Z7
Intrauterine fetal demise	656·4*, V27·1, V27·3, V27·4, V27·6, V27·7	O36·4*, Z37·1, Z37·3, Z37·4, Z37·6, Z37·7
Intrauterine growth restriction	656.5*	O36.5*
Peripartum infection	646·5*, 646·6*, 658·4, 659·3*, 670*, 672*, 674·1*, 674·2*, 674·3*	O23·0*, O23·1*, O23·2*, O23·3*, O23·4*, O41·1*, O75·3*, O85*, O86·0*, O86·1*, O86·2*, O86·4*, O86·8*, O90·0, O90·1
Placental abruption	641·2*	O45*
Post-partum hemorrhage	666*	O67·0, O67·8, O67·9, O72·0, O72·1, O72·2, O72·3
Preterm delivery	644·2	O60·1*
Preeclampsia	642·4*, 642·5*, 642·7*	O11*, O14*
Preterm premature rupture of membranes	658.1	O42·01*, O42·11*, O42·91*
Pulmonary edema[a]	518·4, 428·1, 428·0, 428·21, 428·23, 428·31, 428·33, 428·41, 428·43	J81·0, I50·1, I50·20, I50·21, I50·23, I50·30, I50·31, I50·33, I50·40, I50·41, I50·43, I50·9
Sepsis[a]	O38·*, 995·91, 995·92, 670·2*	O85, O86·04, T80·211, T81·4*
Severe anesthesia complications[a]	668·0*, 668·1*, 668·2*	O74·0, O74·1, O74·2, O74·3, O89·01, O89·09, O89·1, O89·2
Shock[a]	669·1*, 785·5*, 99·0, 995·4, 998·0*	O75·1, R57·*, R65·21, T78·2*, T88·2*, T88·6*, T81·1*
Temporary tracheostomy[a]	PR: 31·1	PR: 0B110Z4, 0B110F4, 0B113Z4, 0B113F4, 0B114Z4, 0B114F4
Transfusion	DX: V58·2<br>PR: 99·0*	PR: 30230H* 30230N* 30230P* 30233H* 30233N* 30233P* 30240H* 30240N* 30240P* 30243H* 30243N* 30243P*
Venous thromboembolism	415·1*, 453·4*, 453·82, 453·83, 453·84, 453·85, 453·86, 453·87, 453·89, 671·3, 671·4, 673·2	I26*, O22·3*, O87·1, O88·2*
Ventilation[a]	PR: 93·90, 96·01, 96·02, 96·03, 96·05	PR: 5A1935Z, 5A1945Z, 5A1955Z

[a]Included in the Severe Maternal Morbidity Index; *Includes all codes that have additional digits, as long as digits preceding the asterisk match

© 2023 Early ML et al. *JAMA Network Open.*

eTable 2. Comparing Code Definitions for Prenatal Anemia vs Published Data

	**Published values[1]**	**All anemia of pregnancy**	**Only deficiency anemia**	**Mixed definition**
Rate in total pregnant population, %	6	13	1	3
**Among pregnant people identified as having anemia**				
Age, yrs, median (IQR)	26·2 (20·2-32·4)	27·7 (27·6-27·7)	27·7 (27·6-27·8)	27·7 (27·6-27·8)
Black race, %	33	25	26	25
Hispanic ethnicity, %	13	22	21	22
Private insurance, %	48	42	41	42
SMM, all 21 indicators, %	8	7	8	6
SMM, no transfusion, %	2	2	3	2
Transfusion, %	7	5	6	5
Postpartum hemorrhage, %	14	8	8	4
Cesarean delivery, %	37	39	38	37

[1] Harrison RK, Lauhon SR, Colvin ZA, McIntosh JJ. Maternal anemia and severe maternal morbidity in a US cohort. Am J Obstet Gynecol MFM. 2021 Sep 1;3(5).

*Note: Severe Maternal Morbidity (SMM)

© 2023 Early ML et al. *JAMA Network Open.*

eTable 3. Proportion of Missing Data by Variable

Variable	Missing among SCD deliveries	Missing among anemia deliveries	Missing among control deliveries	Missing among deliveries, total
Income quartile by zip code, %	1·4	1·0	1·4	1·3
Public insurance, %	0·1	0·1	0·2	0·2
Hospital volume, %	--	--	--	0
Hospital location, %	--	--	--	0
Hospital teaching status, %	--	--	--	0
Hospital ownership, %	--	--	--	0

© 2023 Early ML et al. *JAMA Network Open.*

eTable 4. Survey-Weighted Rates of Composite Severe Maternal Morbidity and Component Events in Delivery Admissions by Group

Complication	Rate, SCD, %	Rate, Anemia, %	Rate, control, %
Severe maternal morbidity	5·90	2·10	1·10
Acute myocardial infarction	0·03	0·02	0·01
Acute pulmonary edema	0·75	0·34	0·12
Acute renal failure	1·21	0·49	0·21
Acute respiratory distress syndrome	1·79	0·34	0·14
Air or thrombotic embolism	0·68	0·16	0·05
Amniotic fluid embolism	--	<0·01	<0·01
Anesthesia complications	0·13	0·03	0·01
Aneurysm	--	--	<0[a]01
Cardiac arrest	0·20	0·02	0·02
Cardioversion	0·13	0·04	0·02
Cerebrovascular event	1·01	0·09	0·05
Disseminated intravascular coagulation	0·01	0·37	0·29
Eclampsia	0·36	0·19	0·13
Hysterectomy	0·29	0·21	0·13
Mechanical ventilation	0·49	0·14	0·07
Obstetric shock	0·36	0·17	0·09
Sepsis	1·27	0·37	0·14
Temporary tracheostomy	0·07	0·01	<0·01

© 2023 Early ML et al. *JAMA Network Open.*
