# Supplementary material for: Comparisons of Severe Maternal Morbidity and Other Adverse Pregnancy Outcomes in Pregnant People With Sickle Cell Disease vs Anemia
Source: JAMA Netw Open. 2023 Feb 2;6(2):e2254545. doi: 10.1001/jamanetworkopen.2022.54545 (PMC9896269; doi:10.1001/jamanetworkopen.2022.54545)
Supplement: Supplement 2. — Data Sharing Statement [file jamanetwopen-e2254545-s002.pdf]

Data Sharing Statement

Early. Comparisons of Severe Maternal Morbidity and Other Adverse Pregnancy Outcomes in Pregnant People With Sickle Cell Disease vs Anemia. JAMA Netw Open. Published online February 2, 2023. doi:10.1001/jamanetworkopen.2022.54545

Data
Data available: No

Additional Information
Explanation for why data not available: The dataset is available through United States federal agencies; however, the data require data use training prior to access.

© 2023 Early ML et al. *JAMA Network Open*.
